# Creation and Validation of a Polysocial Score for Mortality Among Community-Dwelling Older Adults in the United States

**DOI:** 10.1093/geroni/igab046.624

**Published:** 2021-12-17

**Authors:** Chenkai Wu, Michelle Odden, Robert Stawski, Hoda Magid

**Affiliations:** 1 Duke Kunshan University, Kunshan, Jiangsu, China (People's Republic); 2 Stanford University, Stanford, California, United States; 3 Oregon State University, Corvallis, Oregon, United States; 4 Stanford University, Stanford University, California, United States

## Abstract

The interrelatedness between social determinants of health impedes researchers to identify important social factors for health investment. Since the older population had highly diverse social backgrounds, a new approach is needed to quantify the aggregate effect of social factors and develop person-centered social interventions. Participants ([n = 7383], 54.5% female) were aged 65 years or above who complete an additional psychosocial questionnaire in the Health and Retirement Study (HRS) at study entry in 2006 or 2008. Social determinants of health encompassing five social domains: economic stability, neighborhood and physical environment, education, community and social context, and health care system. Five-year mortality was calculated as the number of years from the interview date to the death date. We used the forward stepwise logistic regression to construct the polysocial score and multivariate logistic regressions to assess the associations between polysocial score and five-year mortality. Polysocial score (range: 7 to 59, mean±SD: 35.5±7.5) was created using 15 social determinants of health. Of the 7383 participants, 491 (30.8%), 599 (17.2%), and 166 (7.8%) deaths occurred over five years among participants with a low (0-29), intermediate (30-39), and high (40+) polysocial score, respectively. Participants with an intermediate (Odds Ratio [OR]=0.76; 95% CI, 0.65-0.89) or high (OR=0.46; 95% CI, 0.36-0.59) polysocial score had higher odds of death than those in the low category in the fully adjusted model, respectively. The polysocial approach may offer possible solutions to monitor social environments and suggestions for older adults to improve their social status for specific health outcomes.

